# A Multilevel Framework for the Promotion of Maternal Mental Health and Well-Being During the Perinatal Period

**DOI:** 10.1177/26884844251386007

**Published:** 2025-10-08

**Authors:** Jenn A. Leiferman, Jessica Walls Wilson, Charlotte V. Farewell, Chelsea Walker-Mao, James F. Paulson

**Affiliations:** ^1^ Rocky Mountain Prevention Research Center, Colorado School of Public Health, University of Colorado at the Anschutz Medical Campus, Aurora, Colorado, USA.; ^2^ Department of Community and Behavioral Health, Colorado School of Public Health, University of Colorado at the Anschutz Medical Campus, Aurora, Colorado, USA.; ^3^ Department of Psychology, Old Dominion University, Norfolk, Virginia, USA.

**Keywords:** perinatal, depression, anxiety, well-being, positive psychology, prevention

## Abstract

**Background:**

Depression and anxiety are prevalent in the perinatal period and may contribute to adverse maternal and child health outcomes. A focus on multilevel protective factors may be effective in promoting well-being and increasing quality of life (QOL) in the perinatal period while simultaneously reducing or preventing depression and anxiety symptoms. We tested a conceptual model that posits specific personal, social, and community factors affecting depression, anxiety, and QOL among pregnant and postpartum individuals.

**Methods:**

Four hundred and thirty-eight pregnant or postpartum individuals completed a 121-item cross-sectional survey composed of validated tools that assessed multilevel protective factors, health behaviors, and mental health outcomes. Confirmatory factor analysis (CFA) was constructed to evaluate a 3-part conceptual model against measured indicators of well-being. A structural equation model (SEM) was then fit to test the pattern of associations between our latent structure and three end points: depression, anxiety, and QOL.

**Results:**

The CFA fit the data well (comparative fit index [CFI] = 0.907, Tucker–Lewis index [TLI] = 0.876, root-mean-square error of approximation [RMSEA] = 0.092) supporting the proposed conceptual approach to measuring well-being. The SEM fit well (CFI = 0.964, TLI = 0.941, RMSEA = 0.059), and all three end points were predicted in the hypothesized direction. Social factors predicted reduced anxiety symptomatology, personal and community resources predicted reduced depressive symptomatology, and social resources predicted increased QOL. In addition, we found that collective community resources were associated with social and personal resources, and social resources directly affected personal resources, showing the benefit of multiple levels.

**Conclusions:**

Our findings suggest that both the collection of and interplay between certain personal, social, and community-level resources may prevent and protect against depression and anxiety and promote QOL. Our conceptual model provides a framework to inform future interventions and clinical practice to better assess and promote maternal well-being during the perinatal period.

## Introduction

Depression and anxiety are prevalent during the perinatal period (*i.e.,* pregnancy throughout 1 year postpartum). Approximately 20% of pregnant individuals experience depression, and up to 40% report an anxiety disorder.^1–5^ The most common risk factors are personal history of a mood disorder,^6^ exposure to life stress,^7^ lack of social support,^8,9^ a history of adverse childhood experiences,^10^ marital or partner dissatisfaction,^11^ previous pregnancy loss,^12,13^ and medical complications.^14,15^ Untreated perinatal depression and anxiety are associated with high morbidity and mortality among mothers and their offspring, resulting in a significant economic toll, mainly attributed to reduced productivity among affected mothers and associated adverse maternal outcomes such as cardiovascular disease,^16,17^ hypertension,^18^ and preeclampsia.^19–21^ In addition, untreated depression during pregnancy may increase risk of preterm birth and low birth weight,^22–24^ interrupted bonding between mother and infant,^25,26^ and detrimental growth and development outcomes from early childhood through adolescence,^27,28^ including delayed cognitive development and behavioral problems.^29^

Perinatal depression and anxiety may be treated pharmacologically, including medications such as selective serotonin reuptake inhibitors,^30,31^ which are considered relatively safe^32^ and effective treatment methods.^33,34^ Cognitive behavioral therapy^35,36^ as well as mind–body interventions are efficacious, nonpharmacological treatments for depression and anxiety during the perinatal period.^37,38^ However, these options are often limited due to socioeconomic barriers such as wealth inequality,^1,7^ limited access to adequate prenatal care,^39,40^ cultural factors and stigma that may prevent women from seeking treatment for perinatal mood disorders,^41,42^ perceptions of symptoms,^43,44^ and interpersonal distrust between patients and providers.^45,46^

The transition to motherhood may present unique stressors; however, women who are able to mobilize and utilize a combination of resources that foster their ability to effectively cope report more positive mental health and well-being during the perinatal period.^47–50^ Available resources may be garnered from a combination of system levels.^48^ The socioecological framework is a conceptual model that illustrates an individual surrounded by various systems, namely the microsystem, which contains social influences, and the mesosystem, which consists of community-level influences.^51^ Although there are additional progressively distal systems, our research focuses on the individual, the social, and the community resources that may be categorized by the socioecological model.

**FIG. 1.**
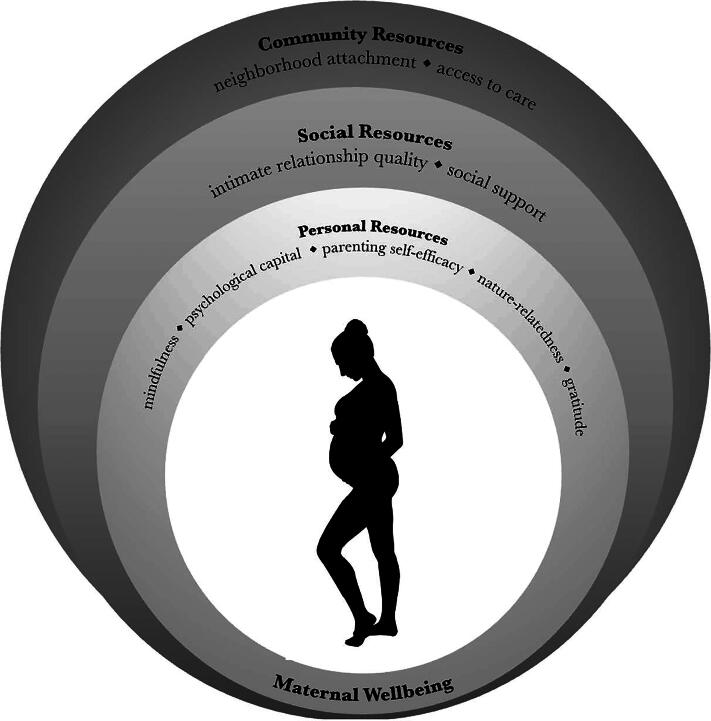
Multilevel conceptual model to promote perinatal mental health and well-being.

In our proposed conceptual model (Fig. 1), personal resources encompass beliefs, attitudes, and behaviors, including mindfulness,^28,37,52^ psychological capital,^53^ gratitude,^54^ self-efficacy,^55^ and nature relatedness.^56^ Social resources include influences through social support,^57–59^ including but not limited to a satisfactory and quality partnership.^60,61^ Community-level resources include access to adequate and quality care^62,63^ and resources available within a community’s built environment such as neighborhood attachment and cohesion.^64^

The purpose of this study was to identify the collection of and the interplay among multilevel resources that can be modified using evidence-based strategies to promote well-being during the perinatal period. We conducted a cross-sectional survey of validated items measuring protective factors across personal, social, and community levels among pregnant and postpartum individuals to test and refine our proposed conceptual model based on extant literature to promote perinatal mental health and well-being.

## Methods

All study procedures were approved by the Colorado Multiple Institutional Review Board (IRB No. 23-0272).

### Recruitment

Recruitment of participants was completed through Centiment, an external company which provides panel recruitment to reach broad and representative audiences for the completion of survey research.^65^ Individuals who were currently pregnant or had given birth within the last 12 weeks and were residing in Colorado were eligible to participate in the survey. Participants were recruited *via* advertisements and directed to Centiment’s website to complete an eligibility screener. Eligible participants subsequently completed an informed consent form followed by the survey. Participants were given a monetary incentive to complete the survey and were entered into a drawing for one of two $50 gift cards. All identifying information (email addresses) was obtained, classified, and discarded by Centiment and was not released to any members of the investigative team. To ensure genuine and accurate data, an “attention question” was placed near the middle of the survey. Those who answered incorrectly (indicating they were not reading the questions, but simply “checking boxes”) were directed to a termination link, and their data were not included in the final sample. All participation took place in January 2023. Data were compiled by Centiment using REDCap,^66^ and then de-identified data were sent to the research team. SPSS^67^ and Mplus^68^ were used to conduct data analyses.

### Survey

The final survey resulted in 121 items comprising validated items related to sociodemographic characteristics, protective factors across personal, social, and community levels, health behaviors, and mental health outcomes, described below.

#### Personal resources

The Mindful Attention Awareness Scale-5 (MAAS-5)^69^ shows high internal validity (α = 0.92). Item analysis demonstrated that a 5-item scale shows the same results as the full survey or the 15-item survey which measures mindful awareness in daily activities (Nos. 7, 8, 9, 10, 14). The Compound PsyCap Scale (CPC-12)^70^ measures psychological capital, which is the combination of self-efficacy, hope, optimism, happiness, and resilience. It measures all strength factors as well as the interplay between them. The Me as a Parent Short Form (MaaPs)^71^ (α = 0.80) is a shortened, 4-item version of the 16-item measure of parental self-efficacy and self-management that can be completed by parents or caretakers of children and measures perceptions of their parenting role. The Gratitude Questionnaire-6 (GQ-6)^72^ is a six-item self-report questionnaire with high reliability (α = 0.82–0.87) designed to assess individual differences in the proneness to experience gratitude in daily life. The GQ-6 is also positively related to optimism, hope, spirituality, and religiousness. The single-item Sleep Quality Scale (SQS)^73^ was developed as a simple and practical sleep quality assessment as an alternative to the Pittsburgh Sleep Quality Index with similar internal validity (α = 0.76–0.92). The Short Form Nature Relatedness Scale (NR-6)^74^ (α = 0.75) assesses the level to which one is connected to nature and how it may contribute to well-being. The survey also included self-report items to assess the frequency of health behaviors like physical activity, sleep, vitamin intake, alcohol consumption, and tobacco and cannabis use, but these were not included in the modeling due to the lack of robustness of single-item measurement.

#### Social resources

To assess relationship status, participants were simply asked “are you in a relationship.” The Relationship Quality Index (RQI-6)^75^ is a 6-item measure designed to assess the quality of relationships in married and cohabiting couples. The Multidimensional Scale of Perceived Social Support (MSPSS)^76^ shows an internal consistency of 0.90–0.94 in a prenatal sample.^77^ To assess religiosity, one statement was presented from the 2006 Benchmark Social Capital Survey^78^ (“My religion gives me a sense of who I am”) which participants were asked to rate from “not at all” to “very much.”

#### Community resources

The Revised Residential Environment Assessment Tool is a reliable, easy-to-use instrument that assesses items 127–132 from a multidimensional questionnaire for the measurement of perceived residential environment quality.^79^ These items specifically measure neighborhood attachment and have a high validity (α = 0.91) when administered separately from the comprehensive scale. The Personal Social Capital Scale-8^80^ (α = 0.83) is used for assessing personally owned social capital, including trustworthy and reciprocal relationships or partnerships, which leads to bridging and bonding to create a network.

#### Mental health and well-being outcomes

The Patient Health Questionnaire-8 (PHQ-8)^81^ (α = 0.85) is a widely used validated diagnostic measure for depressive disorders. It shows high validity and reliability when using a cutoff score of 10. The Generalized Anxiety Disorder-7 (GAD-7)^82^ scale is a brief measure of anxiety. When applied to a prenatal population, it shows high reliability (α = 0.89). When using a cutoff score of 7 or higher, the GAD-7 yielded a sensitivity of 73.3% and a specificity of 67.3%. Quality of life (QOL) was measured by self-reported Likert-type ratings of overall health, physical health, ability to carry out daily activities, absence of fatigue, and absence of pain. Items were identically scaled and were then standardized after being combined into a single QOL scale.

### Data analyses

Each instrument was scored according to scoring instructions provided by its developer, and scores were standardized to z-scores for subsequent modeling. Variables were screened for floor and ceiling effects as well as distribution, with no significant issues identified. We fit an initial confirmatory factor analysis (CFA) model that positioned each of the proposed protective factors within its respective conceptual domain: personal, social, and community resources. After small model refinements were made, this measurement model was used to predict anxiety (GAD-7), depression (PHQ-8), and QOL in three separate models. Because these models examined the role that each of our factors played in predicting these correlates, we limited our models to z-score indicators for each scale rather than item-level indicator entry. Each model used Full-Information Maximum Likelihood estimation to avoid case loss due to missing data. Fit was assessed using comparative fit index (CFI), Tucker–Lewis index (TLI), and root-mean-square error of approximation (RMSEA). Based on initial model fit, we allowed for conceptually driven model specification that was also informed by modification indices.

## Results

A total of 438 participants responded to some items on the survey. Because the survey allowed participants to complete the survey without responding to some items, we found that 360 respondents (90.9%) completed most items, with missingness ranging from 0% to 10.1% across items. Missing items were found to be missing completely at random (Little’s MCAR test chi square = 61.623, *p* = 0.994). For descriptive statistics, we used all available data for reporting. For structural equation model (SEM)-based analyses, we used Full-Information Maximum Likelihood estimation to utilize all available data for modeling.

The average age of the women who participated in the survey was 28.6 years. Of the sample, 20.4% identified as Hispanic. Of the sample, 67.1% identified as Caucasian, 22.4% as African American, and 10.5% as American Indian, Asian, Pacific Islander, or something other than these categories. Of the sample, 9.1% had less than a high school diploma, 34.7% completed high school, 29.9% had some college education, and 26.4% completed college or beyond. The majority of the sample (57.9%) was married or in a domestic partnership, 6.7% were divorced, separated, or widowed, and 35.4% were single. Of the sample, 41.3% were pregnant with or had just had their first baby, and 48.7% had other children (Table 1).

**Table 1.** Participant Demographics (*N* = 438^a^)

	*n*	%
Ethnicity		
Hispanic	85	20.4
Not Hispanic	332	79.6
Race		
African American	94	22.4
American Indian/Asian/Pacific Islander/other	59	10.5
Caucasian	282	67.1
Education		
Less than high school	38	9.1
Completed high school	146	34.7
Some college	126	29.9
Completed college or beyond	111	26.4
Marital status		
Single	148	35.4
Divorced/separated/widowed/other	28	6.7
Married/domestic partner	242	57.9
Parity		
Primiparous	170	41.3
Multiparous	242	48.7
Age	M = 28.58	SD = 6.34

^a^ Participants were not required to answer every question. Therefore, although 438 total women completed the survey, the demographics shown in Table 1 reflect the numbers and percentages of respondents for each category.

SD, standard deviation.

### Confirmatory factor analysis

**FIG. 2.**
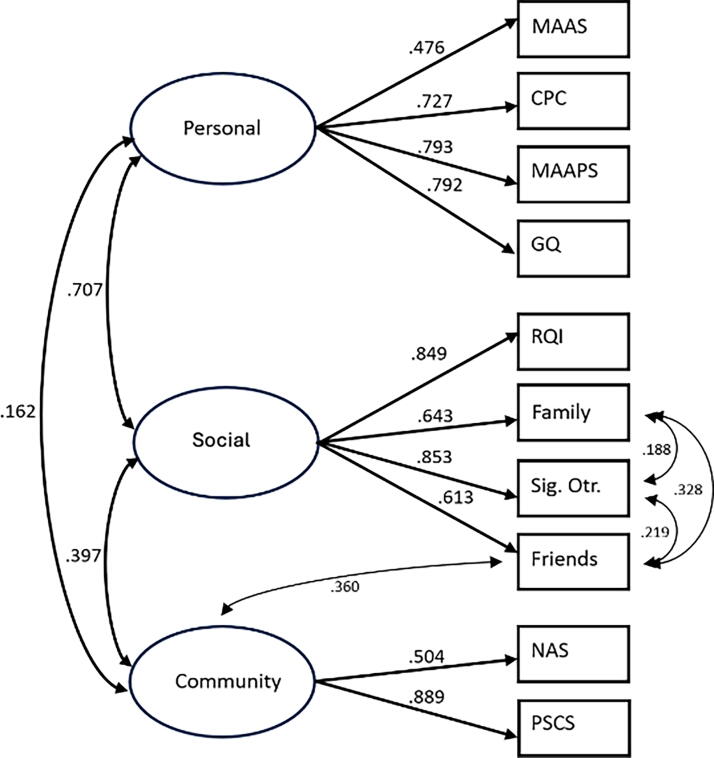
Confirmatory factor analysis for community, social, and personal resources. MAAS, mindfulness; CPC, psychological capital; MAAPS, parenting self-efficacy; GQ, gratitude; RQI, relationship quality; family, family-related social support; Sig. Otr., significant-other-related social support; friends, friend-related social support; NAS, neighborhood attachment; PSCS, personal social capital.

A CFA was constructed to include the following indicators of conceptual latent variables as follows: Personal Resources was measured by Mindfulness, Psychological Capital, Parenting Self-Efficacy, Nature Relatedness, and Gratitude (Fig. 2). Social Resources was measured by Intimate Relationship Quality and Social Support from Family, Friends, and Significant Others. Community Resources was measured by Neighborhood Attachment and Personal Access to Community Resources. This initial model had marginal fit (CFI = 0.907, TLI = 0.876, RMSEA = 0.092), with the low correlations between Nature Relatedness and other constructs contributing substantially to this lack of fit. The model was respecified to remove Nature Relatedness and to allow for error correlations among the Friends, Family, and Significant Other subscales of the MSPSS since these all came from the same instrument. Moreover, the Friends subscale of the MSPSS demonstrated significant error variance correlations with the Community latent variable, so we estimated this error correlation as well. The respecified model demonstrated adequate fit (CFI = 0.963, TLI = 0.938, RMSEA = 0.071), and we moved forward to external correlate models with this as our base exogenous portion of the model.

### Model predicting anxiety, depression, and QOL

**FIG. 3.**
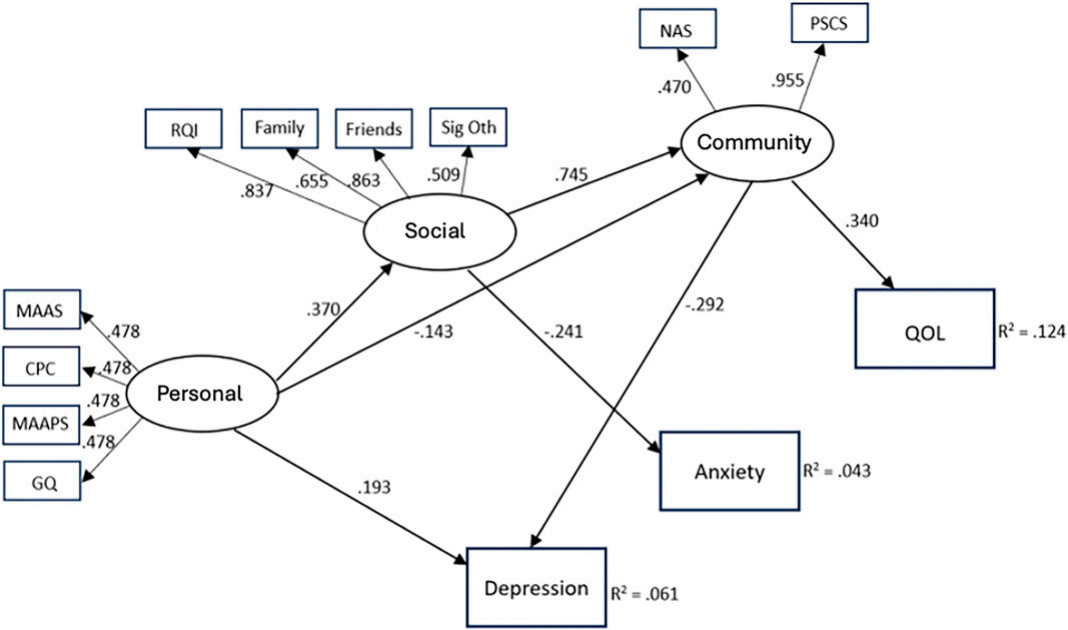
Structural equation modeling predicting depression, anxiety, and quality of life with community, social, and personal resources. All coefficients displayed are standardized XY. Nonsignificant paths are not shown.

A SEM extended our base CFA in two ways (Fig. 3). First, the pattern of influence represented in our social–ecological model was modeled to test direct paths from community factors to social and personal factors, as well as a direct path from social to personal factors. This was done to capture the nesting structure in the model and to enable testing of indirect effects on our end points. Second, the model added three endogenous end point indicators for depression (PHQ-8), anxiety (GAD-7), and QOL. This expanded model demonstrated good fit with the data (CFI = 0.964, TLI = 0.941, RMSEA = 0.059).

In this model, anxiety was negatively predicted directly by the Social Resources latent variable (b = −0.241; *p* = 0.020) such that higher values of Social Resources corresponded with lower levels of anxiety. Anxiety was not directly predicted by the Personal or Community Resources latent variables. We also observed a statistically significant indirect effect, such that Community Resources were positively associated with Social Resources, with Social Resources being negatively associated with Anxiety (b = −0.089, *p* = 0.040).

In this model, depression was negatively predicted directly by the Social Resources latent variable (b = −0.292; *p* = 0.004) such that higher values of Social Resources corresponded with lower levels of depression, but was positively predicted by Community Resources (b = 0.193; *p* = 0.002), which counterintuitively suggests that higher values of Community Resources were associated with higher levels of depression. Depression was not directly predicted by the Personal Resources. We also observed a statistically significant indirect effect, such that Community Resources were positively associated with Social Resources, with Social Resources being negatively associated with Depression (b = −0.108, *p* = 0.018).

In this model, QOL was positively and directly predicted by Personal Resources (b = 0.340; *p* ≤ 0.001) such that higher values of Personal Resources were associated with higher levels of QOL. QOL was not predicted by Community or Social Resources. We also observed a statistically significant indirect effect, such that Social Resources were positively associated with Personal Resources, with Personal Resources being positively associated with QOL (b = 0.253; *p* = 0.001).

## Discussion

Our results support our proposed conceptual model. We found that the model fit well, with all three outcomes being predicted in the hypothesized direction. In particular, social resources predicted anxiety, personal and community resources predicted depression, and social resources predicted QOL.

### Personal resources

Personal resources may aggregate and interact during the perinatal period to collectively influence prenatal, birth, and postpartum outcomes.^48,83^ Our results suggest that personal resources may be protective against depression. This finding is consistent with extant literature suggesting that mindfulness, self-compassion, and psychological capital may enhance maternal well-being and reduce the risk of perinatal mood disorders. In particular, extensive studies have investigated the impacts of mindfulness on perinatal mood disorders and found mindfulness to be protective for perinatal mental health and well-being.^37,52^ A recent systematic review found that maternal participation in mindfulness-based interventions was also associated with reduced perinatal anxiety of moderate-to-large magnitude.^28^ Regular practice of mindfulness promotes self-compassion,^84,85^ a feeling of being kind and understanding toward oneself, rather than inflicting self-criticism.^85,86^ Self-compassion often enhanced with mindfulness practice is also correlated with significantly lower levels of anxiety and depression.^84,85^

Mindfulness practice may also help to harbor the tenants of psychological capital: optimism, hope, self-efficacy, and resilience. Optimism can be described as “a general sense that good things will happen”^52^ and has been shown to be inversely correlated with prenatal depression.^53^ In addition, optimism in pregnancy is associated with fewer depressive symptoms during the postpartum period.^54^ Optimism can impact a woman’s overall health through the promotion of health behaviors such as substance use cessation, conscious healthy eating, physical activity,^87^ and consistent participation in mind–body practices during pregnancy.^88^ Optimism may also contribute to engagement in problem-focused coping strategies^54,87^ and is often used as a modifiable factor in the design of interventions and treatments for perinatal mood disorders.^53^ Hope, much like optimism, is connected to the general belief of good things happening and is often related to the quality of prenatal care received.^89^ Self-efficacy, while a strong indicator of well-being in the general population, takes on a more complex meaning when applied to parenthood; it is protective in itself while also a contributing factor to confidence and relationship satisfaction^55^ as well as social connection, which was evident in our preliminary findings. Resilience, the ability to “bounce back” from difficult situations, is an important strength to foster in the case of difficult, high risk,^90^ or unexpected pregnancies as well as adjusting to new family dynamics.^91^ Access to nature and the presence of green space, including within the built environment, can be protective for maternal mental health. Social prescriptions in which health care providers or community health workers (CHWs) refer an individual to outdoor, health-promoting activities have been shown to promote overall well-being.^56^ Nature-based social prescriptions can encourage women in the perinatal period, especially those in urban settings, to improve their mental health by “prescribing” outdoor activity.^92^ Spending time outdoors can promote physical activity and relieve psychophysiological health,^93^ especially among women living in low-resourced neighborhoods.^94^ Furthermore, some studies link access to green space with more desirable birth outcomes, including birth weight, which can reduce risk for maternal depression and anxiety.^95^

### Social resources

Our study found that social resources are directly impacted by community resources, and social resources directly affect personal resources, confirming our prediction of the directional benefits of a multidimensional model. We found that social resources are specifically protective against anxiety, which reflects extant research.

Partner support is negatively associated with stress in the perinatal period, especially when the mother has risk factors for mood disorders.^60,96^ Marriage or domestic partnership is also protective against adverse birth outcomes such as low birth weight and preterm birth.^97^ Having a partner may also increase health behaviors such as smoking cessation, diet, and physical activity.^97^ Satisfaction within a relationship is also protective. Greater relationship satisfaction is negatively associated with perceived stress.^98^ Furthermore, relationship satisfaction can increase self-efficacy and, therefore, boost well-being for both parents.^61^ Evidence shows that women with higher perceived support from their partner during pregnancy and the postpartum period have decreased depression and anxiety.^99^ This suggests that interventions may benefit from inclusion of partners or referrals to couples therapy if the relationship is less than satisfactory.

As our research shows, social support gained from family members,^100^ peers,^101,102^ and friends is also protective. Perceived social support bolsters perinatal well-being^57^ and also protects against perinatal depression and anxiety.^58^ During stressful life events such as childbirth and the transition to motherhood, women are at an increased risk of perceiving inadequate social support and desire stronger support systems from partners, family, and peers.^103^ Perceived social support during these critical periods mitigates the risk of depression for up to 12 months postpartum.^59^ In addition, recent research shows that an increase in one source of support (*i.e.,* partner, peer, or family) leads to an increase in other sources.^104^

### Community resources

Our results do not reflect the effects of individual constructs within the community level on well-being, but rather show that they collectively affect personal and social levels, as well as collectively show a negative association with anxiety. Taken together, the direct path between Social Resources and Depression and the indirect path that passes from Community Resources through Social Resources may have left little residual variance for the community resources factor, leading to an unusual and counterintuitive finding that merits further exploration.

Access to quality prenatal care, including referrals to specialists and resources for high-risk individuals, is critical for salubrious maternal and child outcomes.^105–107^ Women’s beliefs and behaviors may be influenced by prenatal health providers *via* the appraisal and informational support they provide.^108,109^ Women who value and trust the opinion of their health care providers are more likely to adhere to perinatal care guidelines related to self-care such as diet, exercise, and sleep recommendations.^110,111^ Respectful care also includes autonomy, the ability and confidence to make decisions about their pregnancy and birth.^112^ Structured support in the form of community offerings such as parenting classes and support groups may encompass all levels of resources, as social connections may be made and relationships made stronger, and strategies taught may strengthen personal constructs. Finally, as quality prenatal care is often not equitably accessible,^62^ there is a highlighted need for CHWs such as *promotoras*, doulas, and other community-based leaders to provide support for families navigating the perinatal period and beyond.^105–107,113,114^

The built environment (*e.g.,* structural factors within a community) also influences maternal well-being. Strong neighborhood integration and cohesion may decrease stress and promote positive coping by providing a social network, supportive community, and access to instrumental resources.^115,116^ Low neighborhood cohesion within low-resourced communities creates a compound effect increasing social isolation and exacerbating maternal stress and depression.^117^ Increasing social contact and trust among neighbors reduces fear and protects well-being, especially among residents in low-income neighborhoods, and provides social support that is lacking in family or friends.^118^ Community interventions designed for low-income families that provide opportunities for parents to connect with peers in their community have been successful in well-being promotion.^119^ Specifically, connections between parents are often protective and foster peer advice, support, and connections to other community resources.^120^ This model of community intervention could be applied specifically to the perinatal population to promote instrumental support and resource acquisition.

Our conceptual model proposes that women may benefit by garnering resources across multiple levels to promote their well-being during the perinatal period. Network connections promote the interaction of these personal, social, and community resources. Social capital refers to resources which are embedded in social networks.^121^ This occurs through two network pathways: bonding and bridging. Network connections that promote bonding provide direct emotional and appraisal support by members within a pregnant individual’s personal network (*e.g.,* partner, family, and close friends). In addition to partner and family support, shared experiences in the form of peer interaction enhance bonding and help mitigate mood disorders among pregnant women, demonstrating the need for community-based facilitation of structured peer support during the perinatal period.^122,123^ Multiple “layers” of various types of support are more likely to be achieved related to the number of people a woman in the perinatal period considers to be in her network,^124^ and research shows that the number of ways a person feels connected can cumulatively protect their mental health.^125^ The concept of “bridging” to promote social capital includes the expansion of one’s network beyond family and close friends to accessing community resources. More diverse social networks lead to greater access to information and community resources and, in turn, promote physical^126^ and mental health.^122,123^

## Conclusion

The perinatal period can be a stressful transitional time for many women. Identifying effective resources to promote well-being and decrease the risk of perinatal mood disorders during this time is essential. Screening for risk factors alone may overlook potentially beneficial resources and opportunities for utilizing strengths. Our preliminary research shows directional effects on collective, multilevel protective factors, highlighting the importance of assessing and addressing perinatal mental health and well-being on multiple levels, and underscores the importance of connecting women to protective resources to promote perinatal well-being. More research is needed to better understand the interplay across these proposed pathways and resulting mechanisms that translate to improved maternal well-being during the perinatal period. This understanding can further inform the development of strengths-based screening instruments and protocols to connect women to existing multilevel, evidence-based resources that promote well-being during a critical time.

## Abbreviations Used

CFA: Confirmatory factor analysis
CFI: Comparative fit index
CHW: Community health worker
QOL: Quality of life
RMSEA: Root-mean-square error of approximation
SEM: Structural equation model
TLI: Tucker-Lewis index

## References

[1] Hahn-Holbrook J, Cornwell-Hinrichs T, Anaya I. Economic and health predictors of National postpartum depression prevalence: A systematic review, meta-analysis, and meta-regression of 291 studies from 56 countries. Front Psychiatry 2017;8:248; doi: 10.3389/fpsyt.2017.00248 29449816 PMC5799244

[2] Goodman JH, Watson GR, Stubbs B. Anxiety disorders in postpartum women: A systematic review and meta-analysis. J Affect Disord 2016;203:292–331; doi: 10.1016/j.jad.2016.05.033 27317922

[3] Field T. Postnatal anxiety prevalence, predictors and effects on development: A narrative review. Infant Behav Dev 2018;51:24–32; doi: 10.1016/j.infbeh.2018.02.005 29544195

[4] Yin X, Sun N, Jiang N, et al. Prevalence and associated factors of antenatal depression: Systematic reviews and meta-analyses. Clin Psychol Rev 2021;83:101932; doi: 10.1016/j.cpr.2020.101932 33176244

[5] Shorey S, Chee CYI, Ng ED, et al. Prevalence and incidence of postpartum depression among healthy mothers: A systematic review and meta-analysis. J Psychiatr Res 2018;104:235–248; doi: 10.1016/j.jpsychires.2018.08.001 30114665

[6] Johansen SL, Stenhaug BA, Robakis TK, et al. Past psychiatric conditions as risk factors for postpartum depression: A nationwide cohort study. J Clin Psychiatry 2020;81(1):19m12929. 10.4088/JCP.19m12929 31967747

[7] Lefmann T, Combs-Orme T. Prenatal stress, poverty, and child outcomes. Child Adolesc Soc Work J 2014;31(6):577–590.

[8] Hetherington E, McDonald S, Williamson T, et al. Social support and maternal mental health at 4 months and 1 year postpartum: Analysis from the all our families cohort. J Epidemiol Community Health 2018;72(10):933–939. 29921649 10.1136/jech-2017-210274

[9] Biaggi A, Conroy S, Pawlby S, et al. Identifying the women at risk of antenatal anxiety and depression: A systematic review. J Affect Disord 2016;191:62–77. 26650969 10.1016/j.jad.2015.11.014 PMC4879174

[10] Racine N, Zumwalt K, McDonald S, et al. Perinatal depression: The role of maternal adverse childhood experiences and social support. J Affect Disord 2020;263:576–581. 31759669 10.1016/j.jad.2019.11.030

[11] Kızılırmak A, Calpbinici P, Tabakan G, et al. Correlation between postpartum depression and spousal support and factors affecting postpartum depression. Health Care Women Int 2021;42(12):1325–1339. 32407210 10.1080/07399332.2020.1764562

[12] Rich D. Psychological impact of pregnancy loss: Best practice for obstetric providers. Clin Obstet Gynecol 2018;61(3):628–636. 29596074 10.1097/GRF.0000000000000369

[13] Shaohua L, Shorey S. Psychosocial interventions on psychological outcomes of parents with perinatal loss: A systematic review and meta-analysis. Int J Nurs Stud 2021;117:103871. 33548593 10.1016/j.ijnurstu.2021.103871

[14] Abrar A, Fairbrother N, Smith AP, et al. Anxiety among women experiencing medically complicated pregnancy: A systematic review and meta‐analysis. Birth 2020;47(1):13–20; doi: 10.1111/birt.12443 31222840

[15] Bayrampour H, Vinturache A, Hetherington E, et al. Risk factors for antenatal anxiety: A systematic review of the literature. J Reprod Infant Psychol 2018;36(5):476–503. 30293441 10.1080/02646838.2018.1492097

[16] Ray JG, Vermeulen MJ, Schull MJ, et al. Cardiovascular health after maternal placental syndromes (CHAMPS): Population-based retrospective cohort study. Lancet 2005;366(9499):1797–1803; doi: 10.1016/s0140-6736(05)67726-4 16298217

[17] Auger N, Potter BJ, Healy-Profitós J, et al. Mood disorders in pregnant women and future cardiovascular risk. J Affect Disord 2020;266:128–134; doi: 10.1016/j.jad.2020.01.055 32056866

[18] Shay M, MacKinnon AL, Metcalfe A, et al. Depressed mood and anxiety as risk factors for hypertensive disorders of pregnancy: A systematic review and meta-analysis. Psychol Med 2020;50(13):2128–2140. 32912348 10.1017/S0033291720003062

[19] Howard LM, Khalifeh H. Perinatal mental health: A review of progress and challenges. World Psychiatry 2020;19(3):313–327. 32931106 10.1002/wps.20769 PMC7491613

[20] Moran PS, Wuytack F, Turner M, et al. Economic burden of maternal morbidity–A systematic review of cost-of-illness studies. PLoS One 2020;15(1):e0227377. 31945775 10.1371/journal.pone.0227377 PMC6964978

[21] Bauer A, Knapp M, Parsonage M. Lifetime costs of perinatal anxiety and depression. J Affect Disord 2016;192:83–90. 26707352 10.1016/j.jad.2015.12.005

[22] Grigoriadis S, Graves L, Peer M, et al. Maternal anxiety during pregnancy and the association with adverse perinatal outcomes: Systematic review and meta-analysis. J Clin Psychiatry 2018;79(5):17r12011–17r12010; doi: 10.4088/JCP.17r12011 30192449

[23] Stein A, Pearson RM, Goodman SH, et al. Effects of perinatal mental disorders on the fetus and child. Lancet 2014;384(9956):1800–1819. 25455250 10.1016/S0140-6736(14)61277-0

[24] Bussières E-L, Tarabulsy GM, Pearson J, et al. Maternal prenatal stress and infant birth weight and gestational age: A meta-analysis of prospective studies. Developmental Review 2015;36:179–199; doi: 10.1016/j.dr.2015.04.001

[25] Dubber S, Reck C, Müller M, et al. Postpartum bonding: The role of perinatal depression, anxiety and maternal–fetal bonding during pregnancy. Arch Womens Ment Health 2015;18(2):187–195; doi: 10.1007/s00737-014-0445-4 25088531

[26] Rocha NACF, dos Santos Silva FP, Dos Santos MM, et al. Impact of mother–infant interaction on development during the first year of life: A systematic review. J Child Health Care 2020;24(3):365–385. 31337225 10.1177/1367493519864742

[27] Blair MM, Glynn LM, Sandman CA, et al. Prenatal maternal anxiety and early childhood temperament. Stress 2011;14(6):644–651; doi: 10.3109/10253890.2011.594121 21790468 PMC10486312

[28] Shi Z, MacBeth A. The effectiveness of mindfulness-based interventions on maternal perinatal mental health outcomes: A systematic review. Mindfulness (N Y) 2017;8(4):823–847; doi: 10.1007/s12671-016-0673-y 28757900 PMC5506176

[29] Slomian J, Honvo G, Emonts P, et al. Consequences of maternal postpartum depression: A systematic review of maternal and infant outcomes. Womens Health (Lond) 2019;15:1745506519844044. 31035856 10.1177/1745506519844044 PMC6492376

[30] Molenaar NM, Bais B, Lambregtse-van den Berg MP, et al. The international prevalence of antidepressant use before, during, and after pregnancy: A systematic review and meta-analysis of timing, type of prescriptions and geographical variability. J Affect Disord 2020;264:82–89. 31846905 10.1016/j.jad.2019.12.014

[31] Marchesi C, Ossola P, Amerio A, et al. Clinical management of perinatal anxiety disorders: A systematic review. J Affect Disord 2016;190:543–550. 26571104 10.1016/j.jad.2015.11.004

[32] Bellantuono C, Martellini M, Orsolini L. General approach to pharmacological treatment: during the perinatal period. In: Perinatal Psychopharmacology. Springer: 2019; pp. 55–66.

[33] Jarde A, Morais M, Kingston D, et al. Neonatal outcomes in women with untreated antenatal depression compared with women without depression: A systematic review and meta-analysis. JAMA Psychiatry 2016;73(8):826–837. 27276520 10.1001/jamapsychiatry.2016.0934

[34] Nillni YI, Mehralizade A, Mayer L, et al. Treatment of depression, anxiety, and trauma-related disorders during the perinatal period: A systematic review. Clin Psychol Rev 2018;66:136–148. 29935979 10.1016/j.cpr.2018.06.004 PMC6637409

[35] Shortis E, Warrington D, Whittaker P. The efficacy of cognitive behavioral therapy for the treatment of antenatal depression: A systematic review. J Affect Disord 2020;272:485–495. 32553392 10.1016/j.jad.2020.03.067

[36] Sockol LE. A systematic review of the efficacy of cognitive behavioral therapy for treating and preventing perinatal depression. J Affect Disord 2015;177:7–21. 25743368 10.1016/j.jad.2015.01.052

[37] Dhillon A, Sparkes E, Duarte RV. Mindfulness-based interventions during pregnancy: A systematic review and meta-analysis. Mindfulness (N Y) 2017;8(6):1421–1437; doi: 10.1007/s12671-017-0726-x 29201244 PMC5693962

[38] Lever Taylor B, Cavanagh K, Strauss C. The effectiveness of mindfulness-based interventions in the perinatal period: A systematic review and meta-analysis. PLoS One 2016;11(5):e0155720; doi: 10.1371/journal.pone.0155720 27182732 PMC4868288

[39] Shaw E, Levitt C, Wong S, et al.; McMaster University Postpartum Research Group. Systematic review of the literature on postpartum care: Effectiveness of postpartum support to improve maternal parenting, mental health, quality of life, and physical health. Birth 2006;33(3):210–220. 16948721 10.1111/j.1523-536X.2006.00106.x

[40] Smith MS, Lawrence V, Sadler E, et al. Barriers to accessing mental health services for women with perinatal mental illness: Systematic review and meta-synthesis of qualitative studies in the UK. BMJ Open 2019;9(1):e024803. 10.1136/bmjopen-2018-024803 PMC6347898 30679296

[41] Bodnar-Deren S, Benn E, Balbierz A, et al. Stigma and postpartum depression treatment acceptability among black and white women in the first six-months postpartum. Matern Child Health J 2017;21(7):1457–1468; doi: 10.1007/s10995-017-2263-6 28102504

[42] Goodman SH, Dimidjian S, Williams KG. Pregnant African American women’s attitudes toward perinatal depression prevention. Cultur Divers Ethnic Minor Psychol 2013;19(1):50–57; doi: 10.1037/a0030565 23356356

[43] Mathur VA, Morris T, McNamara K. Cultural conceptions of Women’s labor pain and labor pain management: A mixed-method analysis. Soc Sci Med 2020;261:113240; doi: 10.1016/j.socscimed.2020.113240 32758799

[44] Rosenthal L, Overstreet NM, Khukhlovich A, et al. Content of, sources of, and responses to sexual stereotypes of Black and Latinx women and men in the United States: A qualitative intersectional exploration. J Soc Issues 2020;76(4):921–948; doi: 10.1111/josi.12411

[45] Webb R, Uddin N, Ford E, et al.; MATRIx study team. Barriers and facilitators to implementing perinatal mental health care in health and social care settings: A systematic review. Lancet Psychiatry 2021;8(6):521–534. 33838118 10.1016/S2215-0366(20)30467-3

[46] Grand-Guillaume-Perrenoud JA, Origlia P, Cignacco E. Barriers and facilitators of maternal healthcare utilisation in the perinatal period among women with social disadvantage: A theory-guided systematic review. Midwifery 2022;105:103237. 34999509 10.1016/j.midw.2021.103237

[47] Lazarus RS, Folkman S. Transactional theory and research on emotions and coping. Eur J Pers 1987;1(3):141–169.

[48] Hobfoll SE. Social and psychological resources and adaptation. Review of General Psychology 2002;6(4):307–324.

[49] Latendresse G. The interaction between chronic stress and pregnancy: Preterm birth from a biobehavioral perspective. J Midwifery Womens Health 2009;54(1):8–17. 19114234 10.1016/j.jmwh.2008.08.001 PMC2651684

[50] Freche RE. II., Optimism and resilience, as moderated by coping style, on prenatal depressive symptomology and salivary cortisol response to stress. California State University, Long Beach: 2013.

[51] Bronfenbrenner U. Toward an experimental ecology of human development. Am Psychol 1977;32(7):513–531.

[52] Corno G, Espinoza M, Maria Baños R. A narrative review of positive psychology interventions for women during the perinatal period. J Obstet Gynaecol 2019;39(7):889–895; doi: 10.1080/01443615.2019.1581735 31179814

[53] Evans EC, Bullock LF. Optimism and other psychosocial influences on antenatal depression: A systematic review. Nurs Health Sci 2012;14(3):352–361. 22762538 10.1111/j.1442-2018.2012.00700.x

[54] Grote NK, Bledsoe SE. Predicting postpartum depressive symptoms in new mothers: The role of optimism and stress frequency during pregnancy. Health Soc Work 2007;32(2):107–118. 17571644 10.1093/hsw/32.2.107

[55] Wittkowski A, Garrett C, Calam R, et al. Self-Report Measures of Parental Self-Efficacy: A Systematic Review of the Current Literature. J Child Fam Stud 2017;26(11):2960–2978; doi: 10.1007/s10826-017-0830-5 29081640 PMC5646137

[56] Bickerdike L, Booth A, Wilson PM, et al. Social prescribing: Less rhetoric and more reality. A systematic review of the evidence. BMJ Open 2017;7(4):e013384. 10.1136/bmjopen-2016-013384 PMC5558801 28389486

[57] Wadephul F, Glover L, Jomeen J. Conceptualising women’s perinatal well-being: A systematic review of theoretical discussions. Midwifery 2020;81:102598. 31835103 10.1016/j.midw.2019.102598

[58] Bedaso A, Adams J, Peng W, et al. The relationship between social support and mental health problems during pregnancy: A systematic review and meta-analysis. Reprod Health 2021;18(1):162–123. 34321040 10.1186/s12978-021-01209-5 PMC8320195

[59] Hagaman A, LeMasters K, Zivich PN, et al. Longitudinal effects of perinatal social support on maternal depression: A marginal structural modelling approach. J Epidemiol Community Health 2021;75(10):936–943. 33712512 10.1136/jech-2020-215836 PMC8434957

[60] Stapleton LRT, Schetter CD, Westling E, et al. Perceived partner support in pregnancy predicts lower maternal and infant distress. J Fam Psychol 2012;26(3):453–463. 22662772 10.1037/a0028332 PMC3992993

[61] Wynter K, Rowe H, Fisher J. Interactions between perceptions of relationship quality and postnatal depressive symptoms in Australian, primiparous women and their partners. Aust J Prim Health 2014;20(2):174–181. 23622442 10.1071/PY12066

[62] Hajizadeh S, Ramezani Tehrani F, Simbar M, et al. Factors influencing the use of prenatal care: A systematic review. Journal of Midwifery and Reproductive Health 2016;4(1):544–557.

[63] Sword W, Heaman MI, Brooks S, et al. Women’s and care providers’ perspectives of quality prenatal care: A qualitative descriptive study. BMC Pregnancy Childbirth 2012;12(1):29–18. 22502640 10.1186/1471-2393-12-29 PMC3352181

[64] Witten K, Kearns R, McCreanor T, et al. Connecting place and the everyday practices of parenting: Insights from Auckland, New Zealand. Environ Plan A 2009;41(12):2893–2910.

[65] Centiment. Survey with Centiment. 2024. Available from: https://www.centiment.co

[66] Harris PA, Taylor R, Thielke R, et al. Research electronic data capture (REDCap)—a metadata-driven methodology and workflow process for providing translational research informatics support. J Biomed Inform 2009;42(2):377–381; doi: 10.1016/j.jbi.2008.08.010 18929686 PMC2700030

[67] Verma J. Data analysis in management with SPSS software. Springer Science & Business Media: 2012.

[68] Muthén B. Applications of causally defined direct and indirect effects in mediation analysis using SEM in Mplus. Los Angeles, CA: 2011.

[69] Brown KW, Ryan RM. The benefits of being present: Mindfulness and its role in psychological well-being. J Pers Soc Psychol 2003;84(4):822–848. 12703651 10.1037/0022-3514.84.4.822

[70] Lorenz T, Beer C, Pütz J, et al. Measuring psychological capital: Construction and validation of the compound PsyCap scale (CPC-12). PLoS One 2016;11(4):e0152892. 27035437 10.1371/journal.pone.0152892 PMC4817957

[71] Hamilton VE, Matthews JM, Crawford SB. Development and preliminary validation of a parenting self-regulation scale: “Me as a Parent”. J Child Fam Stud 2015;24(10):2853–2864; doi: 10.1007/s10826-014-0089-z

[72] McCullough ME, Emmons RA, Tsang J-A. The grateful disposition: A conceptual and empirical topography. J Pers Soc Psychol 2002;82(1):112–127. 11811629 10.1037//0022-3514.82.1.112

[73] Snyder E, Cai B, DeMuro C, et al. A new single-item sleep quality scale: Results of psychometric evaluation in patients with chronic primary insomnia and depression. J Clin Sleep Med 2018;14(11):1849–1857. 30373688 10.5664/jcsm.7478 PMC6223557

[74] Nisbet EK, Zelenski JM. The NR-6: A new brief measure of nature relatedness. Front Psychol 2013;4:813; doi: 10.3389/fpsyg.2013.00813 24198806 PMC3814587

[75] Early Intervention Foundation. Relationship Quality Index (RQI). 2020.

[76] Zimet GD, Dahlem NW, Zimet SG, et al. The multidimensional scale of perceived social support. J Pers Assess 1988;52(1):30–41.

[77] Zimet GD, Powell SS, Farley GK, et al. Psychometric characteristics of the multidimensional scale of perceived social support. J Pers Assess 1990;55(3–4):610–617. 2280326 10.1080/00223891.1990.9674095

[78] Research; RCFPO. 20065 Social Capital Community Benchmark Survey. 2006.

[79] Bonaiuto M, Aiello A, Perugini M, et al. Multidimensional perception of residential environment quality and neighborhood attachment in the urban environment. J Environ Psychol 1999;19(4):331–352; doi: 10.1006/jevp.1999.0138

[80] Wang P, Chen X, Gong J, et al. Reliability and validity of the personal social capital scale 16 and personal social capital scale 8: Two short instruments for survey studies. Soc Indic Res 2014;119(2):1133–1148; doi: 10.1007/s11205-013-0540-3

[81] Kroenke K, Strine TW, Spitzer RL, et al. The PHQ-8 as a measure of current depression in the general population. J Affect Disord 2009;114(1–3):163–173. 18752852 10.1016/j.jad.2008.06.026

[82] Spitzer RL, Kroenke K, Williams JB, et al. A brief measure for assessing generalized anxiety disorder: The GAD-7. Arch Intern Med 2006;166(10):1092–1097; doi: 10.1001archinte.166.10.1092 16717171 10.1001/archinte.166.10.1092

[83] Dunkel Schetter C. Psychological science on pregnancy: Stress processes, biopsychosocial models, and emerging research issues. Annu Rev Psychol 2011;62:531–558. 21126184 10.1146/annurev.psych.031809.130727

[84] Felder JN, Lemon E, Shea K, et al. Role of self-compassion in psychological well-being among perinatal women. Arch Womens Ment Health 2016;19(4):687–690. 27138783 10.1007/s00737-016-0628-2

[85] Townshend K, Caltabiano N. Self-compassion and mindfulness: Modeling change processes associated with the reduction of perinatal depression. J Child Fam Stud 2019;28(7):1790–1802; doi: 10.1007/s10826-019-01371-2

[86] Neff KD, Kirkpatrick KL, Rude SS. Self-compassion and adaptive psychological functioning. J Res Pers 2007;41(1):139–154.

[87] Gill RM, Loh JM. The role of optimism in health-promoting behaviors in new primiparous mothers. Nurs Res 2010;59(5):348–355. 20697308 10.1097/NNR.0b013e3181ed6b11

[88] Reis PJ, Alligood MR. Prenatal yoga in late pregnancy and optimism, power, and well-being. Nurs Sci Q 2014;27(1):30–36. 24403034 10.1177/0894318413509706

[89] Hoseini ES, Rahmati R, Shaghaghi F, et al. The relationship between hope and happiness with prenatal care. J Educ Health Promot 2020;9:206; doi: 10.4103/jehp.jehp_141_20 33062739 PMC7530412

[90] Ilska M, Przybyła-Basista H. The role of partner support, ego-resiliency, prenatal attitudes maternity and pregnancy in psychological well-being of women in high-risk and low-risk pregnancy. Psychol Health Med 2020;25(5):630–638. 32151169 10.1080/13548506.2020.1737718

[91] Serpeloni F, Radtke KM, Hecker T, et al. Does prenatal stress shape postnatal resilience?–an epigenome-wide study on violence and mental health in humans. Front Genet 2019;10:269. 31040859 10.3389/fgene.2019.00269 PMC6477038

[92] Leavell M, Leiferman J, Gascon M, et al. Nature-based social prescribing in urban settings to improve social connectedness and mental well-being: A review. Curr Environ Health Rep 2019;6(4):297–308. 31713144 10.1007/s40572-019-00251-7

[93] Dadvand P, de Nazelle A, Figueras F, et al. Green space, health inequality and pregnancy. Environ Int 2012;40:110–115. 21824657 10.1016/j.envint.2011.07.004

[94] McEachan R, Prady S, Smith G, et al. The association between green space and depressive symptoms in pregnant women: Moderating roles of socioeconomic status and physical activity. J Epidemiol Community Health 2016;70(3):253–259. 26560759 10.1136/jech-2015-205954 PMC4789818

[95] Agay-Shay K, Peled A, Crespo AV, et al. Green spaces and adverse pregnancy outcomes. Occup Environ Med 2014;71(8):562–569; doi: 10.1136/oemed-2013-101961 24759971

[96] Galbally M, Watson SJ, Boyce P, et al. The role of trauma and partner support in perinatal depression and parenting stress: An Australian pregnancy cohort study. Int J Soc Psychiatry 2019;65(3):225–234. 30915877 10.1177/0020764019838307

[97] Kane JB. Marriage advantages in perinatal health: Evidence of marriage selection or marriage protection? J Marriage Fam 2016;78(1):212–229. 26778858 10.1111/jomf.12257 PMC4712954

[98] Tissera H, Auger E, Séguin L, et al. Happy prenatal relationships, healthy postpartum mothers: A prospective study of relationship satisfaction, postpartum stress, and health. Psychol Health 2021;36(4):461–477. 32449394 10.1080/08870446.2020.1766040

[99] Dennis CL, Ross L. Women’s perceptions of partner support and conflict in the development of postpartum depressive symptoms. J Adv Nurs 2006;56(6):588–599. 17118038 10.1111/j.1365-2648.2006.04059.x

[100] Razurel C, Kaiser B, Antonietti J-P, et al. Relationship between perceived perinatal stress and depressive symptoms, anxiety, and parental self-efficacy in primiparous mothers and the role of social support. Women Health 2017;57(2):154–172. 26909523 10.1080/03630242.2016.1157125

[101] Leger J, Letourneau N. New mothers and postpartum depression: A narrative review of peer support intervention studies. Health Soc Care Community 2015;23(4):337–348. 25346377 10.1111/hsc.12125

[102] Dennis C-L. Postpartum depression peer support: Maternal perceptions from a randomized controlled trial. Int J Nurs Stud 2010;47(5):560–568. 19962699 10.1016/j.ijnurstu.2009.10.015

[103] Razurel C, Bruchon-Schweitzer M, Dupanloup A, et al. Stressful events, social support and coping strategies of primiparous women during the postpartum period: A qualitative study. Midwifery 2011;27(2):237–242. 19783333 10.1016/j.midw.2009.06.005

[104] Racine N, Plamondon A, Hentges R, et al. Dynamic and bidirectional associations between maternal stress, anxiety, and social support: The critical role of partner and family support. J Affect Disord 2019;252:19–24. 30954841 10.1016/j.jad.2019.03.083

[105] Perriman N, Davis DL, Ferguson S. What women value in the midwifery continuity of care model: A systematic review with meta-synthesis. Midwifery 2018;62:220–229. 29723790 10.1016/j.midw.2018.04.011

[106] Chang Y-S, Coxon K, Portela AG, et al. Interventions to support effective communication between maternity care staff and women in labour: A mixed-methods systematic review. Midwifery 2018;59:4–16. 29351865 10.1016/j.midw.2017.12.014 PMC5852259

[107] Pais M, Pai MV. Stress among pregnant women: A systematic review. J Clin Diagn Res 2018;12(5):1–4.

[108] Kominiarek MA, O’Dwyer LC, Simon MA, et al. Targeting obstetric providers in interventions for obesity and gestational weight gain: A systematic review. PLoS One 2018;13(10):e0205268. 30289912 10.1371/journal.pone.0205268 PMC6173456

[109] Santo EC, Forbes PW, Oken E, et al. Determinants of physical activity frequency and provider advice during pregnancy. BMC Pregnancy Childbirth 2017;17(1):286–211. 28870169 10.1186/s12884-017-1460-z PMC5583983

[110] Downe S, Lawrie TA, Finlayson K, et al. Effectiveness of respectful care policies for women using routine intrapartum services: A systematic review. Reprod Health 2018;15(1):23–13. 29409519 10.1186/s12978-018-0466-y PMC5801845

[111] Ferrari RM, Siega-Riz AM, Evenson KR, et al. A qualitative study of women’s perceptions of provider advice about diet and physical activity during pregnancy. Patient Educ Couns 2013;91(3):372–377. 23399436 10.1016/j.pec.2013.01.011 PMC3683874

[112] Vedam S, Stoll K, Martin K, et al.; Changing Childbirth in BC Steering Council. The Mother’s Autonomy in Decision Making (MADM) scale: Patient-led development and psychometric testing of a new instrument to evaluate experience of maternity care. PLoS One 2017;12(2):e0171804. 28231285 10.1371/journal.pone.0171804 PMC5322919

[113] Bill DE, Hock-Long L, Mesure M, et al. Healthy start programa madrina: A promotora home visiting outreach and education program to improve perinatal health among Latina pregnant women. Health Educator 2009;41(2):68–76.

[114] Hans SL, Edwards RC, Zhang Y. Randomized controlled trial of doula-home-visiting services: Impact on maternal and infant health. Matern Child Health J 2018;22 (Suppl 1):105–113. 29855838 10.1007/s10995-018-2537-7 PMC6153776

[115] M. Franco L, J. Pottick K, Huang CC. Early parenthood in a community context: Neighborhood conditions, race–ethnicity, and parenting stress. Journal Community Psychology 2010;38(5):574–590.

[116] Tofani AA, de Almeida Lamarca G, Sheiham A, et al. The different effects of neighbourhood and individual social capital on health-compromising behaviours in women during pregnancy: A multi-level analysis. BMC Public Health 2015;15(1):890–817. 26369830 10.1186/s12889-015-2213-4 PMC4570677

[117] Kohen DE, Leventhal T, Dahinten VS, et al. Neighborhood disadvantage: Pathways of effects for young children. Child Dev 2008;79(1):156–169; doi: 10.1111/j.1467-8624.2007.01117.x 18269515

[118] Kruger DJ, Reischl TM, Gee GC. Neighborhood social conditions mediate the association between physical deterioration and mental health. Am J Community Psychol 2007;40(3–4):261–271; doi: 10.1007/s10464-007-9139-7 17924185

[119] Bess KD, Doykos B. Tied together: Building relational well‐being and reducing social isolation through place‐based parent education. Journal Community Psychology 2014;42(3):268–284.

[120] Plesko CM, Yu Z, Tobin K, et al. Social connectedness among parents raising children in low‐income communities: An integrative review. Res Nurs Health 2021;44(6):957–969. 34647625 10.1002/nur.22189 PMC9292156

[121] Lin N. Building a network theory of social capital. Social Capital 2017:3–28.

[122] Dennis CL, Hodnett E, Kenton L, et al. Effect of peer support on prevention of postnatal depression among high risk women: Multisite randomised controlled trial. Bmj 2009;338:a3064; doi: 10.1136/bmj.a3064 19147637 PMC2628301

[123] Fang Q, Lin L, Chen Q, et al. Effect of peer support intervention on perinatal depression: A meta-analysis. Gen Hosp Psychiatry 2022;74:78–87. 34942447 10.1016/j.genhosppsych.2021.12.001

[124] Razurel C, Kaiser B. The role of satisfaction with social support on the psychological health of primiparous mothers in the perinatal period. Women Health 2015;55(2):167–186. 25775391 10.1080/03630242.2014.979969

[125] Libbey H, Ireland M, Resnick M. Social connectedness: Is protection cumulative? Journal of Adolescent Health 2002;30(2):102.

[126] Cohen S, Janicki-Deverts D. Can we improve our physical health by altering our social networks? Perspect Psychol Sci 2009;4(4):375–378; doi: 10.1111/j.1745-6924.2009.01141.x 20161087 PMC2744289

